# Foodborne Disease Outbreaks Associated with Marine Toxins — Foodborne Disease Outbreak Surveillance System, United States, 2011–2023

**DOI:** 10.15585/mmwr.ss7503a1

**Published:** 2026-06-18

**Authors:** Cassie M. Hartley, Son T. Hoang, Virginia A. Roberts, Anna N. Chard

**Affiliations:** ^1^National Center for Emerging and Zoonotic Infectious Diseases, CDC, Atlanta, Georgia; ^2^Oak Ridge Institute for Science and Education, Oak Ridge, Tennessee

## Abstract

**Problem/Condition:**

Marine toxins cause most of the noninfectious outbreaks reported to CDC’s Foodborne Disease Outbreak Surveillance System (FDOSS) each year. Certain marine toxins are produced by algae that accumulate in aquatic animals through the food chain, whereas others appear as a result of improper food storage and are therefore preventable. Overgrowths of toxin-producing algae (harmful algal blooms) have occurred on both U.S. coasts; the historical geographic range for some species (e.g., *Gambierdiscus* in tropical and subtropical regions) have expanded. Marine toxins that cause foodborne illness are tasteless, odorless, resistant to cooking or freezing, and can produce a complex variety of gastrointestinal, neurologic, and neuropsychologic symptoms. Among persons with severe illness resulting from ingestion of marine toxins, cardiovascular and respiratory manifestations can result in hospitalization and death. Analyzing these outbreaks provides insight into their incidence, severity, and key characteristics, which can be used to guide food safety and foodborne illness prevention efforts.

**Period Covered:**

2011–2023.

**Description of System:**

Via FDOSS, CDC collects data on foodborne disease outbreaks from local, state, and territorial health departments in the United States. Foodborne disease outbreaks are defined as two or more cases of similar illness associated with a common food exposure; outbreak etiologies and food sources can be reported as confirmed or suspected. Since 2009, health departments have voluntarily reported foodborne disease outbreaks to FDOSS through CDC’s web-based National Outbreak Reporting System.

**Results:**

During 2011–2023, a total of 402 foodborne disease outbreaks caused by marine toxins were reported to FDOSS. These outbreaks resulted in 1,280 illnesses, 96 hospitalizations, and one death. The national rate of these reported outbreaks was 1.2 outbreaks per 1 million population. Outbreaks were reported by 32 states; Washington, DC; and Puerto Rico. Hawaii (25.3 outbreaks per 1 million population), Puerto Rico (16.5), Florida (6.3), and Alaska (5.4) had the highest reported rates. A food source was identified in 396 (99%) outbreaks, of which 379 (96%) implicated fish. Among 313 outbreak investigations in which the food importation status was known, 219 (70%) of the implicated foods were not imported. Of the 377 outbreaks in which a single location of food preparation was identified, private homes were reported in 193 (51%) outbreaks and sit-down dining restaurants were reported in 130 (34%) outbreaks.

Nearly all outbreak reports (95%) implicated scombroid toxin (192 outbreaks, 597 illnesses, and six hospitalizations) or ciguatoxin (189 outbreaks, 619 illnesses, and 67 hospitalizations). For the 192 scombroid toxin outbreaks, the jurisdictions reporting the highest number of outbreaks were New York (43 [22%]), Florida (38 [20%]), California (23 [12%]), and Hawaii (18 [9%]). Of the 189 scombroid toxin outbreak reports with a reported food source, the majority (76%) implicated tuna. Of the 131 scombroid toxin outbreak reports with information on importation status, 70 (53%) implicated imported foods. Of the 181 scombroid toxin outbreak reports with a single location of food preparation, sit-down dining restaurants were identified in 104 (57%) outbreaks.

Of the 189 ciguatoxin outbreaks, Florida reported 88 (47%) outbreaks, Puerto Rico reported 55 (29%), and Hawaii reported 18 (10%). Of the 187 ciguatoxin outbreak reports with a reported food source, 58 (31%) implicated barracuda, 25 (13%) implicated grouper, and 22 (12%) implicated amberjack. The majority (87%) of the 164 ciguatoxin outbreak reports with a known importation status reported foods that were domestically caught. Of the 178 ciguatoxin outbreak reports with a single location of food preparation, private homes were identified in 142 (80%) outbreaks.

Shellfish-associated toxins caused 13 outbreaks, including paralytic shellfish poisoning in six (46%) outbreaks, neurotoxic shellfish poisoning in four (31%) outbreaks, and amnesic shellfish poisoning, diarrhetic shellfish poisoning, and unknown shellfish poisoning each associated with one (8%) outbreak. These outbreaks resulted in 40 illnesses and nine hospitalizations. Florida reported five (38%) of these 13 outbreaks and Alaska reported four (31%). Of the 13 total shellfish-associated outbreaks, mussels were implicated in four outbreaks (31%), sea snails in four (31%), and clams in three (23%). None of these outbreak investigations implicated imported shellfish. Of the 11 shellfish-associated toxin outbreaks with a reported single food preparation location, private homes were identified in eight (73%) outbreaks.

**Interpretation:**

Characterizing marine toxin outbreaks reported to FDOSS can guide opportunities for prevention. Scombroid toxin and ciguatoxin caused the most reported outbreaks, illnesses, and hospitalizations of all marine toxins, indicating they are important targets for public health intervention. More than half of scombroid toxin outbreaks were caused by imported fish and fish prepared in sit-down dining restaurants. Most ciguatoxin outbreaks were caused by reef fish, fish that were not imported, and fish prepared in private homes. Outbreaks attributed to shellfish-associated toxins were caused by shellfish that were not imported and were prepared in private homes. Outbreaks caused by ciguatoxin and those associated with shellfish-associated toxins were predominantly reported by jurisdictions where toxin-producing algal species are endemic and often implicated recreationally caught fish and shellfish. Geographic expansion, increasing frequency, and increasing intensity of harmful algal blooms in U.S. coastal waters might increase the presence of ciguatoxin and shellfish-associated toxins in aquatic animals. The varying characteristics of outbreaks caused by marine toxins highlight the need for tailored prevention measures that account for both environmental conditions and consumer behaviors.

**Public Health Action:**

The findings in this report can be used by public health practitioners to guide food safety prevention efforts and raise awareness about marine toxins and associated illnesses. Prevention of outbreaks resulting from scombroid toxin from both imported and domestic fish involves maintaining temperature control of seafood below 40°F (4.4°C) from catch to consumption. Understanding the needs and practices of recreational harvesters could help public health officials craft targeted communications about safer practices for harvest location and affected aquatic species. Reducing the harvesting of reef fish and shellfish from high-risk areas, especially during and immediately after harmful algal blooms, might prevent illnesses from these toxins.

## Introduction

Marine toxins are consistently the most commonly reported cause of noninfectious foodborne disease outbreaks in the United States (*1*,*2*). Scombroid toxin poisoning occurs after the consumption of high levels of histamine produced in fish that were not properly refrigerated after being caught (*3*). Improper holding and storage of fish at temperatures ≥40°F (≥4.4°C) can result in the production of histamine and other scombroid toxins by bacteria with high histidine carboxylase activity (*4*). Other marine toxins (e.g., ciguatoxin) are produced by algae and accumulate in the flesh of fish and shellfish through the food chain, occur naturally within fish species, or originate from unknown sources (e.g., those causing Haff disease) (*5*,*6*). Marine toxins that cause foodborne illness are tasteless, odorless, and resistant to cooking or freezing, making it challenging to prevent illness once aquatic animals have been contaminated. Illnesses from marine toxins can cause a wide range of nonspecific gastrointestinal and neurologic symptoms, including nausea, vomiting, diarrhea, flushing, numbness and tingling, and cold allodynia (i.e., painful responses to cold stimuli), among others. Less commonly, symptoms might persist for months or years in severe cases, and illnesses can produce cardiovascular and respiratory complications that lead to hospitalization and death (*6*).

Consumption of fish and shellfish in the United States has increased since the 1980s (*3*,*7*). This increasing demand for fish and shellfish might contribute to an increased risk for foodborne disease outbreaks and illnesses from marine toxins. Imported fish and shellfish are estimated to comprise 80% to >90% of all domestically consumed seafood (*8*–*10*). Although certain parts of the supply chain of imported seafood (e.g., processors and certain transporters) are subject to Hazard Analysis Critical Control Point regulations set by the Food and Drug Administration’s Imported Seafood Safety Program, others (e.g., vessels that harvest or transport but do not otherwise process fish) are exempt (*11*), which can introduce opportunities for improper seafood handling practices (*3*,*12*). In addition to nutrient pollution, climate-related changes to marine ecosystems such as warming waters have expanded the geographic distribution of algal species that produce marine toxins, and their blooms have become longer, more frequent, and more intense (*7*,*13*,*14*). These phenomena might increase the presence of toxins in fish and shellfish in the areas where they are commercially and recreationally harvested. The incidence of illness associated with marine toxins is not well characterized in the United States; although national foodborne disease outbreak data include outbreaks caused by marine toxins, national case surveillance for marine and freshwater harmful algal bloom–associated illnesses did not start until 2016 and does not exist for other marine toxins (*15*,*16*).

This report summarizes data on all foodborne disease outbreaks associated with marine toxin etiologies reported to CDC via the Foodborne Disease Outbreak Surveillance System (FDOSS) during 2011–2023, building on analyses of noninfectious foodborne disease exposures during 2000–2010 (*17*) and fish-associated foodborne disease outbreaks during 1998–2015 with data through 2023 (*18*). Although previous publications have focused on case studies, single outbreaks or etiologies, or summaries of illnesses in specific jurisdictions, this report provides the first national summary of all foodborne outbreaks caused by these toxins in the United States. Analyzing these outbreaks can reveal their incidence, severity, and key characteristics. These findings can guide food safety efforts of public health officials and seafood processors and raise awareness of these toxins among health care professionals, recreational fishers, and seafood consumers.

## Methods

### Data Source

In the United States, local, state, and territorial health departments voluntarily report foodborne disease outbreaks to FDOSS, originally developed in 1973 as a paper-based surveillance system; electronic reporting for FDOSS started in 1998. In 2009, CDC began to receive FDOSS reports through the National Outbreak Reporting System (NORS), which expanded national electronic outbreak reporting to encompass foodborne and waterborne disease outbreaks, as well as enteric disease outbreaks caused by contact with animals, infected persons, environmental sources, or unknown modes of transmission.

This summary includes all finalized reports of foodborne disease outbreaks that implicated a marine toxin with a date of first illness onset during January 1, 2011–December 31, 2023. Data for this summary were downloaded from NORS on December 30, 2024. Midyear state, territory, and federal district populations during 2011–2023 were obtained from the U.S. Census Bureau (*19*).

### Definitions

A foodborne disease outbreak is defined as two or more cases of the same illness resulting from the ingestion of a common food. When exposure occurs in a single state, territory, or federal district, the outbreak is categorized as a single-state exposure outbreak; when exposure occurs in two or more states, the outbreak is categorized as a multistate exposure outbreak. Local, state, and territorial health departments classify etiologies as confirmed, suspected, or unknown on the basis of specific criteria for foodborne outbreaks (*20*). Implicated foods are categorized as confirmed or suspected using epidemiologic, laboratory, or traceback evidence; environmental assessment; or other data collected by investigators. If an implicated food is not reported, the food source is classified as unknown.

### Variables

Data summarized for each marine toxin etiology (confirmed and suspected) include counts of outbreaks, illnesses, hospitalizations, and deaths; state, territory, or federal district of exposure; implicated food source (confirmed and suspected); location of preparation of the implicated food; and whether the implicated food was imported from outside of the United States.

Etiologies were categorized as they were reported to FDOSS by local, state, and territorial health departments, except when multiple values could be categorized under a single etiology. This exception applied to scombroid toxin, which includes both scombroid toxin and histamine, and paralytic shellfish poison, which includes both paralytic shellfish poison and saxitoxin. Implicated foods were categorized using the common name for the food reported (e.g., tuna includes foods reported as tuna steak, albacore, ahi, and yellowfin tuna; grouper includes foods reported as red grouper, purplespotted grouper, and roi). If a food source was not reported or was reported as unknown, the implicated food was categorized as “food source not reported or unknown.” Investigators reported the location where the implicated food was prepared from a picklist of 32 location types or included additional locations in a free text field. Similar locations were grouped as indicated (e.g., private home includes locations reported as private home or residence and single-family home). Free-text comment fields were reviewed to determine whether implicated foods were recreationally harvested.

### Data Analysis

This report characterizes all confirmed and suspected marine toxin–associated foodborne disease outbreaks reported during 2011–2023 and finalized in FDOSS as of December 30, 2024. Descriptive analyses were conducted using R software (version 4.4.0; R Foundation). A sensitivity analysis was performed to compare findings derived from outbreaks with confirmed etiologies to outbreaks with both confirmed and suspected etiologies. Population-based outbreak reporting rates for each jurisdiction were calculated using U.S. Census Bureau midyear population estimates. An average of the midyear population estimates for each year was used as the denominator for rate calculations for each jurisdiction. This activity was reviewed by CDC, deemed not research, and conducted consistent with applicable federal law and CDC policy.^§^

## Results

### All Etiologies

During 2011–2023, local, state, and territorial health departments reported 402 outbreaks associated with 10 marine toxin etiologies (371 confirmed and 31 suspected), resulting in 1,280 illnesses, 96 hospitalizations, and one death (Table 1). The median number of outbreaks per year was 30 (range = 11–52) (Supplementary Figure 1). The median number of illnesses per outbreak was two (range = 2–50). The national rate of reported outbreaks during 2011–2023 was 1.2 outbreaks per 1 million population (Figure). Single-state outbreaks were reported by 32 states; Washington, DC; and Puerto Rico; one multistate outbreak was reported and included 11 states. Hawaii (25.3 outbreaks per 1 million population), Puerto Rico (16.5), Florida (6.3), and Alaska (5.4) had the highest rates of reported outbreaks during 2011–2023. Outbreaks reported by these jurisdictions accounted for 56% of outbreaks and 54% of illnesses (Table 2).

**TABLE 1:** Number and percentage* of reported outbreaks and outbreak-associated illnesses, hospitalizations, and deaths, by confirmed and suspected marine toxin etiology — Foodborne Disease Outbreak Surveillance System, United States, 2011–2023

Etiology	No. of outbreaks			No. of illnesses			No. of hospitalizations			No. of deaths		
	Confirmed etiology	Suspected etiology	Total (%)*	Confirmed etiology	Suspected etiology	Total (%)*	Confirmed etiology	Suspected etiology	Total (%)*	Confirmed etiology	Suspected etiology	Total (%)*
**Scombroid toxin**	182	10	**192 (48)**	564	33	**597 (47)**	5	1	**6 (6)**	0	0	**0 (—^†^)**
**Ciguatoxin**	174	15	**189 (47)**	570	49	**619 (48)**	59	8	**67 (70)**	0	0	**0 (—)**
**Shellfish-associated toxin**	9	4	**13 (3)**	30	10	**40 (3)**	7	2	**9 (9)**	0	0	**0 (—)**
Paralytic shellfish poison	5	1	**6 (1)**	21	2	**23 (2)**	4	0	**4 (4)**	0	0	**0 (—)**
Neurotoxic shellfish poison	1	3	**4 (1)**	2	8	**10 (1)**	1	2	**3 (3)**	0	0	**0 (—)**
Amnesic shellfish poison	1	0	**1 (—)**	2	0	**2 (—)**	2	0	**2 (2)**	0	0	**0 (—)**
Diarrhetic shellfish poison	1	0	**1 (—)**	3	0	**3 (—)**	0	0	**0 (—)**	0	0	**0 (—)**
Unknown shellfish poison	1	0	**1 (—)**	2	0	**2 (—)**	0	0	**0 (—)**	0	0	**0 (—)**
**Other marine toxin**	6	2	**8 (2)**	17	7	**24 (2)**	11	3	**14 (15)**	1	0	**1 (100)**
Haff disease (unidentified toxin)	3	1	**4 (—)**	8	3	**11 (1)**	7	3	**10 (10)**	1	0	**1 (100)**
Puffer fish tetrodotoxin	3	0	**3 (1)**	9	0	**9 (1)**	4	0	**4 (4)**	0	0	**0 (—)**
Unknown fish toxin	0	1	**1 (—)**	0	4	**4 (—)**	0	0	**0 (—)**	0	0	**0 (—)**
**Total**	**371**	**31**	**402**	**1,181**	**99**	**1,280**	**82**	**14**	**96**	**1**	**0**	**1**

* The denominator for the percentages is the total number of outbreaks, illnesses, hospitalizations, or deaths, respectively.

^†^ Dashes indicate percentage values <1%.

**FIGURE:**
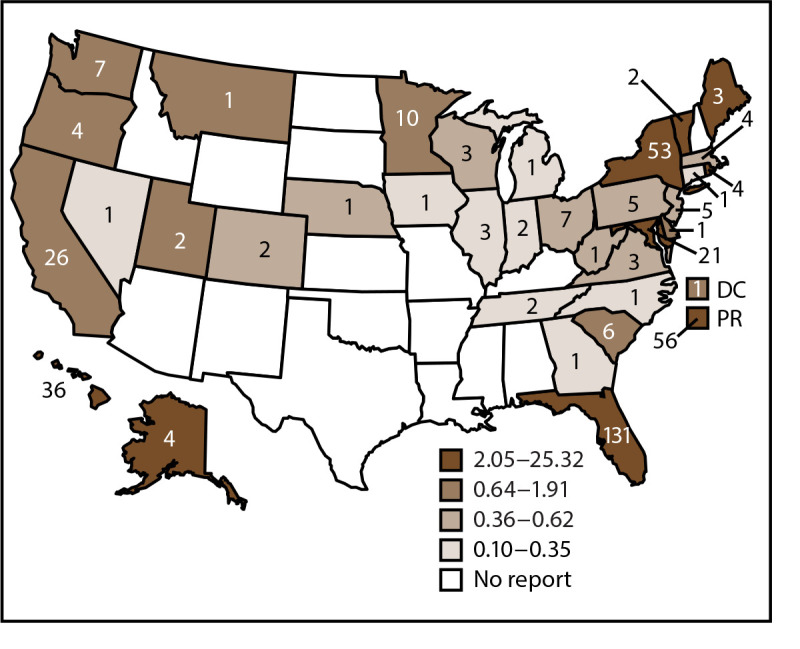
Number of outbreaks* and average annual rate^†^ of reported foodborne disease outbreaks associated with marine toxins, by state, territory, or federal district — Foodborne Disease Outbreak Surveillance System, United States, 2011–2023 * Includes one multistate outbreak (i.e., outbreak in which exposure occurred in more than one state) assigned as one outbreak to each state involved. ^†^ Per 1 million population, based on U.S. Census Bureau (https://data.census.gov) 2011–2023 midyear population estimates, with cut points for outbreak rate categories determined using quartiles.

**TABLE 2:** Number and percentage* of reported marine toxin outbreaks and outbreak-associated illnesses, by state, territory, or federal district of exposure — Foodborne Disease Outbreak Surveillance System, United States, 2011–2023

State, territory, or federal district of exposure	Total		Scombroid toxin		Ciguatoxin		Shellfish-associated toxins^†^		Other marine toxins^§^	
	No. of outbreaks (%)	No. of illnesses (%)	No. of outbreaks (%)	No. of illnesses (%)	No. of outbreaks (%)	No. of illnesses (%)	No. of outbreaks (%)	No. of illnesses (%)	No. of outbreaks (%)	No. of illnesses (%)
Florida	**131 (33)**	**379 (30)**	38 (20)	93 (16)	88 (47)	274 (44)	5 (38)	12 (30)	0 (—^¶^)	0 (—)
Puerto Rico	**56 (14)**	**190 (15)**	1 (1)	5 (1)	55 (29)	185 (30)	0 (—)	0 (—)	0 (—)	0 (—)
New York	**52 (13)**	**125 (10)**	43 (22)	103 (17)	7 (4)	18 (3)	0 (—)	0 (—)	2 (25)	4 (17)
Hawaii	**36 (9)**	**105 (8)**	18 (9)	51 (9)	18 (10)	54 (9)	0 (—)	0 (—)	0 (—)	0 (—)
California	**26 (6)**	**73 (6)**	23 (12)	64 (11)	2 (1)	5 (1)	0 (—)	0 (—)	1 (13)	4 (17)
Maryland	**20 (5)**	**65 (5)**	10 (5)	30 (5)	10 (5)	35 (6)	0 (—)	0 (—)	0 (—)	0 (—)
Minnesota	**9 (2)**	**25 (2)**	8 (4)	23 (4)	0 (—)	0 (—)	0 (—)	0 (—)	1 (13)	2 (8)
Washington	**7 (1)**	**22 (2)**	5 (3)	12 (2)	0 (—)	0 (—)	2 (15)	10 (25)	0 (—)	0 (—)
Ohio	**6 (1)**	**25 (2)**	4 (2)	19 (3)	1 (1)	4 (1)	1 (8)	2 (5)	0 (—)	0 (—)
South Carolina	**6 (1)**	**20 (2)**	4 (2)	13 (2)	2 (1)	7 (1)	0 (—)	0 (—)	0 (—)	0 (—)
Alaska	**4 (1)**	**14 (1)**	0 (—)	0 (—)	0 (—)	0 (—)	4 (31)	14 (35)	0 (—)	0 (—)
Massachusetts	**4 (1)**	**13 (1)**	3 (2)	10 (2)	1 (1)	3 (—)	0 (—)	0 (—)	0 (—)	0 (—)
New Jersey	**4 (1)**	**8 (1)**	3 (2)	6 (1)	0 (—)	0 (—)	1 (8)	2 (5)	0 (—)	0 (—)
Oregon	**4 (1)**	**9 (1)**	3 (2)	7 (1)	1 (1)	2 (—)	0 (—)	0 (—)	0 (—)	0 (—)
Pennsylvania	**4 (1)**	**17 (1)**	2 (1)	7 (1)	1 (1)	5 (1)	0 (—)	0 (—)	1 (13)	5 (21)
Illinois	**3 (1)**	**9 (1)**	1 (1)	2 (—)	0 (—)	0 (—)	0 (—)	0 (—)	2 (25)	7 (29)
Rhode Island	**3 (1)**	**8 (1)**	2 (1)	6 (1)	0 (—)	0 (—)	0 (—)	0 (—)	1 (13)	2 (8)
Virginia	**3 (1)**	**14 (1)**	3 (2)	14 (2)	0 (—)	0 (—)	0 (—)	0 (—)	0 (—)	0 (—)
Wisconsin	**3 (1)**	**15 (1)**	2 (1)	8 (1)	1 (1)	7 (1)	0 (—)	0 (—)	0 (—)	0 (—)
Colorado	**2 (—)**	**12 (1)**	2 (1)	12 (2)	0 (—)	0 (—)	0 (—)	0 (—)	0 (—)	0 (—)
Indiana	**2 (—)**	**7 (—)**	2 (1)	7 (1)	0 (—)	0 (—)	0 (—)	0 (—)	0 (—)	0 (—)
Maine	**2 (—)**	**20 (2)**	2 (1)	20 (3)	0 (—)	0 (—)	0 (—)	0 (—)	0 (—)	0 (—)
Tennessee	**2 (—)**	**5 (—)**	2 (1)	5 (1)	0 (—)	0 (—)	0 (—)	0 (—)	0 (—)	0 (—)
Utah	**2 (—)**	**4 (—)**	2 (1)	4 (1)	0 (—)	0 (—)	0 (—)	0 (—)	0 (—)	0 (—)
Connecticut	**1 (—)**	**2 (—)**	1 (1)	2 (—)	0 (—)	0 (—)	0 (—)	0 (—)	0 (—)	0 (—)
District of Columbia	**1 (—)**	**6 (—)**	0 (—)	0 (—)	1 (1)	6 (1)	0 (—)	0 (—)	0 (—)	0 (—)
Georgia	**1 (—)**	**14 (1)**	0 (—)	0 (—)	1 (1)	14 (2)	0 (—)	0 (—)	0 (—)	0 (—)
Iowa	**1 (—)**	**2 (—)**	1 (1)	2 (—)	0 (—)	0 (—)	0 (—)	0 (—)	0 (—)	0 (—)
Michigan	**1 (—)**	**2 (—)**	1 (1)	2 (—)	0 (—)	0 (—)	0 (—)	0 (—)	0 (—)	0 (—)
Montana	**1 (—)**	**3 (—)**	1 (1)	3 (1)	0 (—)	0 (—)	0 (—)	0 (—)	0 (—)	0 (—)
Nebraska	**1 (—)**	**3 (—)**	1 (1)	3 (1)	0 (—)	0 (—)	0 (—)	0 (—)	0 (—)	0 (—)
Nevada	**1 (—)**	**7 (1)**	1 (1)	7 (1)	0 (—)	0 (—)	0 (—)	0 (—)	0 (—)	0 (—)
North Carolina	**1 (—)**	**4 (1)**	1 (1)	4 (1)	0 (—)	0 (—)	0 (—)	0 (—)	0 (—)	0 (—)
Vermont	**1 (—)**	**3 (—)**	1 (1)	3 (1)	0 (—)	0 (—)	0 (—)	0 (—)	0 (—)	0 (—)
Multistate**	**1 (—)**	**50 (4)**	1 (1)	50 (8)	0 (—)	0 (—)	0 (—)	0 (—)	0 (—)	0 (—)
**Total**	**402**	**1,280**	**192**	**597**	**189**	**619**	**13**	**40**	**8**	**24**

* The denominator for percentages is the total number of outbreaks or illnesses, respectively.

^†^ Outbreaks associated with toxins that cause paralytic shellfish poisoning occurred in Alaska (four outbreaks with 14 illnesses), Washington (one outbreak with seven illnesses), and Florida (one outbreak with two illnesses); neurotoxic shellfish poisoning outbreaks occurred in Florida (four outbreaks with 10 illnesses); an amnesic shellfish poisoning outbreak occurred in New Jersey (one outbreak with two illnesses); a diarrhetic shellfish poisoning occurred in Washington (one outbreak with three illnesses); and an outbreak resulting from an unknown shellfish toxin poisoning occurred in Ohio (one outbreak with two illnesses).

^§^ Outbreaks of Haff disease occurred in Illinois (two outbreaks with seven illnesses) and New York (two outbreaks with four illnesses); puffer fish tetrodotoxin poisoning outbreaks occurred in Pennsylvania (one outbreak with five illnesses), Minnesota (one outbreak with two illnesses), and Rhode Island (one outbreak with two illnesses); and an outbreak resulting from an unknown fish toxin occurred in California (one outbreak with four illnesses).

^¶^ Dashes indicate percentage values <1%.

** Multistate outbreaks are outbreaks in which exposure occurred in more than one state. Exposure states were Ohio (11 illnesses), Vermont (seven), Delaware (six), New York (six), Pennsylvania (six), Maryland (four), Rhode Island (three), Maine (two), Minnesota (two), New Jersey (two), and West Virginia (one).

Nearly all (95%) outbreak investigations implicated scombroid toxin or ciguatoxin (381 outbreaks). Thirteen outbreak investigations (3%) implicated shellfish-associated toxins (Table 1). A single food source was identified in 396 (99%) outbreaks, of which 379 (96%) implicated fish, 12 (3%) implicated shellfish (mussels, clams, and sea snails), and five (1%) implicated other foods (sushi, roe, octopus, and seafood pasta) (Table 3). Of the 313 (78%) outbreak reports that listed information about importation status, the implicated food was not imported for 219 (70%) reports (Table 4). Information about how the implicated food was harvested was available for 157 (39%) outbreaks, of which 42 (27%) indicated the implicated food was recreationally harvested (Table 5). Among the 377 (94%) outbreak investigations that reported a single location of preparation, 193 (51%) were linked to private homes and 147 (39%) were linked to restaurants. Sit-down dining restaurants were reported for 130 (34%) of the 147 restaurant-associated outbreaks (Table 6). The sensitivity analysis comparing outbreaks with confirmed etiologies to those with both confirmed and suspected etiologies did not identify substantial differences in these findings.

**TABLE 3:** Number and percentage* of reported marine toxin outbreaks and outbreak-associated illnesses, by implicated food source — Foodborne Disease Outbreak Surveillance System, United States, 2011–2023

Food source	Total		Scombroid toxin		Ciguatoxin		Shellfish-associated toxins^†^		Other marine toxins^§^	
	No. of outbreaks (%)	No. of illnesses (%)	No. of outbreaks (%)	No. of illnesses (%)	No. of outbreaks (%)	No. of illnesses (%)	No. of outbreaks (%)	No. of illnesses (%)	No. of outbreaks (%)	No. of illnesses (%)
**Fish**	**379 (96)**	**1,205 (96)**	186 (98)	576 (98)	186 (99)	611 (99)	1 (8)	2 (5)	6 (75)	16 (80)
Tuna	**144 (36)**	**476 (38)**	144 (76)	476 (81)	0 (—^¶^)	0 (—)	0 (—)	0 (—)	0 (—)	0 (—)
Barracuda	**58 (15)**	**187 (15)**	0 (—)	0 (—)	58 (31)	187 (31)	0 (—)	0 (—)	0 (—)	0 (—)
Amberjack	**25 (6)**	**106 (8)**	3 (2)	6 (1)	22 (12)	100 (16)	0 (—)	0 (—)	0 (—)	0 (—)
Grouper	**25 (6)**	**81 (6)**	0 (—)	0 (—)	25 (13)	81 (13)	0 (—)	0 (—)	0 (—)	0 (—)
Mahi mahi	**20 (5)**	**48 (4)**	19 (10)	46 (8)	1 (1)	2 (—)	0 (—)	0 (—)	0 (—)	0 (—)
Snapper	**15 (4)**	**41 (3)**	2 (1)	4 (1)	13 (7)	37 (6)	0 (—)	0 (—)	0 (—)	0 (—)
Mackerel	**14 (4)**	**41 (3)**	1 (1)	2 (—)	13 (7)	39 (6)	0 (—)	0 (—)	0 (—)	0 (—)
Jack	**13 (3)**	**45 (4)**	0 (—)	0 (—)	13 (7)	45 (7)	0 (—)	0 (—)	0 (—)	0 (—)
Hogfish	**12 (3)**	**34 (3)**	0 (—)	0 (—)	12 (6)	34 (6)	0 (—)	0 (—)	0 (—)	0 (—)
Puffer fish	**4 (1)**	**11 (1)**	0 (—)	0 (—)	0 (—)	0 (—)	1 (8)	2 (5)	3 (43)	9 (45)
Escolar	**3 (1)**	**10 (1)**	3 (2)	10 (2)	0 (—)	0 (—)	0 (—)	0 (—)	0 (—)	0 (—)
Salmon	**3 (1)**	**6 (—)**	3 (2)	6 (1)	0 (—)	0 (—)	0 (—)	0 (—)	0 (—)	0 (—)
Seabass	**3 (1)**	**11 (1)**	0 (—)	0 (—)	3 (2)	11 (2)	0 (—)	0 (—)	0 (—)	0 (—)
Wahoo	**3 (1)**	**6 (—)**	3 (2)	6 (1)	0 (—)	0 (—)	0 (—)	0 (—)	0 (—)	0 (—)
Buffalo fish	**2 (1)**	**5 (—)**	0 (—)	0 (—)	0 (—)	0 (—)	0 (—)	0 (—)	2 (29)	5 (25)
Kole	**2 (1)**	**6 (—)**	0 (—)	0 (—)	2 (1)	6 (1)	0 (—)	0 (—)	0 (—)	0 (—)
Marlin	**2 (1)**	**7 (1)**	2 (1)	7 (1)	0 (—)	0 (—)	0 (—)	0 (—)	0 (—)	0 (—)
African pompano	**1 (—)**	**3 (—)**	0 (—)	0 (—)	1 (1)	3 (—)	0 (—)	0 (—)	0 (—)	0 (—)
Anchovy	**1 (—)**	**3 (—)**	1 (1)	3 (1)	0 (—)	0 (—)	0 (—)	0 (—)	0 (—)	0 (—)
Bass	**1 (—)**	**2 (—)**	0 (—)	0 (—)	1 (1)	2 (—)	0 (—)	0 (—)	0 (—)	0 (—)
Carp	**1 (—)**	**2 (—)**	0 (—)	0 (—)	0 (—)	0 (—)	0 (—)	0 (—)	1 (14)	2 (10)
Cod	**1 (—)**	**2 (—)**	1 (1)	2 (—)	0 (—)	0 (—)	0 (—)	0 (—)	0 (—)	0 (—)
Eel	**1 (—)**	**4 (—)**	0 (—)	0 (—)	1 (1)	4 (1)	0 (—)	0 (—)	0 (—)	0 (—)
Lionfish	**1 (—)**	**4 (—)**	0 (—)	0 (—)	1 (1)	4 (1)	0 (—)	0 (—)	0 (—)	0 (—)
Monchong	**1 (—)**	**2 (—)**	1 (1)	2 (—)	0 (—)	0 (—)	0 (—)	0 (—)	0 (—)	0 (—)
Sheepshead	**1 (—)**	**2 (—)**	0 (—)	0 (—)	1 (1)	2 (—)	0 (—)	0 (—)	0 (—)	0 (—)
Surgeonfish	**1 (—)**	**2 (—)**	0 (—)	0 (—)	1 (1)	2 (—)	0 (—)	0 (—)	0 (—)	0 (—)
Swordfish	**1 (—)**	**2 (—)**	0 (—)	0 (—)	1 (1)	2 (—)	0 (—)	0 (—)	0 (—)	0 (—)
Tilapia	**1 (—)**	**2 (—)**	1 (1)	2 (—)	0 (—)	0 (—)	0 (—)	0 (—)	0 (—)	0 (—)
Triggerfish	**1 (—)**	**2 (—)**	0 (—)	0 (—)	1 (1)	2 (—)	0 (—)	0 (—)	0 (—)	0 (—)
Multiple**	**3 (1)**	**9 (1)**	1 (1)	2 (—)	2 (1)	7 (1)	0 (—)	0 (—)	0 (—)	0 (—)
Unknown	**15 (4)**	**43 (3)**	3 (2)	6 (1)	14 (7)	41 (7)	0 (—)	0 (—)	0 (—)	0 (—)
**Shellfish**	**12 (3)**	**38 (3)**	1 (—)	2 (—)	0 (—)	0 (—)	11 (85)	36 (90)	0 (—)	0 (—)
Clams	**4 (1)**	**11 (1)**	1 (1)	2 (—)	0 (—)	0 (—)	3 (23)	9 (23)	0 (—)	0 (—)
Mussels	**4 (1)**	**17 (1)**	0 (—)	0 (—)	0 (—)	0 (—)	4 (31)	17 (43)	0 (—)	0 (—)
Sea snails	**4 (1)**	**10 (1)**	0 (—)	0 (—)	0 (—)	0 (—)	4 (31)	10 (25)	0 (—)	0 (—)
**Other**	**5 (1)**	**16 (1)**	2 (1)	8 (1)	1 (—)	2 (—)	1 (8)	2 (5)	1 (13)	4 (20)
Sushi	**2 (1)**	**7 (1)**	1 (1)	5 (1)	1 (1)	2 (—)	0 (—)	0 (—)	0 (—)	0 (—)
Octopus	**1 (—)**	**3 (—)**	1 (1)	3 (1)	0 (—)	0 (—)	0 (—)	0 (—)	0 (—)	0 (—)
Roe	**1 (—)**	**4 (—)**	0 (—)	0 (—)	0 (—)	0 (—)	0 (—)	0 (—)	1 (14)	4 (20)
Seafood pasta	**1 (—)**	**2 (—)**	0 (—)	0 (—)	0 (—)	0 (—)	1 (8)	2 (5)	0 (—)	0 (—)
**Food source reported**	**396 (99)**	**1,259 (98)**	189 (98)	586 (98)	187 (99)	613 (99)	13 (100)	40 (100)	7 (88)	20 (83)
**Food source not reported or unknown**	**6 (1)**	**21 (2)**	3 (2)	11 (2)	2 (1)	6 (1)	0 (—)	0 (—)	1 (13)	4 (17)
**Total**	**402**	**1,280**	**192**	**597**	**189**	**619**	**13**	**40**	**8**	**24**

* The denominator for the food source percentages is the food source reported total. Denominators for percentages of food source reported and food source not reported or unknown are the total.

^†^ Outbreak investigations for toxins associated with paralytic shellfish poison implicated clams (three outbreaks with nine illnesses), mussels (two outbreaks with 12 illnesses), and puffer fish (one outbreak with two illnesses); neurotoxic shellfish poison implicated sea snails (four outbreaks with ten illnesses); diarrhetic shellfish poison implicated mussels (one outbreak with three illnesses); amnesic shellfish poison implicated mussels (one outbreak with two illnesses); and unknown shellfish poison implicated seafood pasta (one outbreak with two illnesses).

^§^ Outbreak investigations for Haff disease implicated buffalo fish (two outbreaks with five illnesses) and carp (one outbreak with two illnesses). One investigation did not identify a food source (four illnesses). Outbreak investigations for puffer fish tetrodotoxin implicated puffer fish (three outbreaks with nine illnesses); investigation of an unknown fish toxin implicated fish roe (one outbreak with four illnesses).

^¶^ Dashes indicate percentage values <1%.

** Amberjack and hogfish (one ciguatoxin outbreak), snapper and mackerel (one ciguatoxin outbreak), and tuna and mahi mahi (one scombroid toxin outbreak).

**TABLE 4:** Number and percentage* of reported marine toxin outbreaks and outbreak-associated illnesses, by importation status of implicated food — Foodborne Disease Outbreak Surveillance System, United States, 2011–2023

Food importation status	Total		Scombroid toxin		Ciguatoxin		Shellfish-associated toxins		Other marine toxins	
	No. of outbreaks (%)	No. of illnesses (%)	No. of outbreaks (%)	No. of illnesses (%)	No. of outbreaks (%)	No. of illnesses (%)	No. of outbreaks (%)	No. of illnesses (%)	No. of outbreaks (%)	No. of illnesses (%)
Imported	**94 (30)**	**327 (32)**	70 (53)	258 (60)	22 (13)	62 (12)	0 (—)	0 (—)	2 (29)	7 (35)
Not imported	**219 (70)**	**692 (68)**	61 (47)	173 (40)	142 (87)	472 (88)	11 (100)	34 (100)	5 (71)	13 (65)
**Subtotal**	**313 (78)**	**1,019 (80)**	131 (68)	431 (72)	164 (87)	534 (86)	11 (85)	34 (85)	7 (88)	20 (83)
Not reported or unknown	**89 (22)**	**261 (20)**	61 (32)	166 (28)	25 (13)	85 (14)	2 (15)	6 (15)	1 (13)	4 (17)
**Total**	**402**	**1,280**	**192**	**597**	**189**	**619**	**13**	**40**	**8**	**24**

* The denominator for percentages of foods with a reported importation status is the subtotal. The denominator for percentages of foods with unknown importation status is the total.

**TABLE 5:** Number and percentage* of reported marine toxin outbreaks and outbreak-associated illnesses, by harvesting practice of implicated food — Foodborne Disease Outbreak Surveillance System, United States, 2011–2023

Harvesting practice	Total		Scombroid toxin		Ciguatoxin		Shellfish-associated toxins		Other marine toxins	
	No. of outbreaks (%)	No. of illnesses (%)	No. of outbreaks (%)	No. of illnesses (%)	No. of outbreaks (%)	No. of illnesses (%)	No. of outbreaks (%)	No. of illnesses (%)	No. of outbreaks (%)	No. of illnesses (%)
Recreational	**42 (27)**	**117 (24)**	0 (—)	0 (—)	36 (60)	99 (56)	6 (86)	18 (90)	0 (—)	0 (—)
Not recreational	**115 (73)**	**374 (76)**	88 (100)	289 (100)	24 (40)	79 (44)	1 (14)	2 (10)	2 (100)	4 (100)
**Subtotal**	**157 (39)**	**491 (38)**	88 (46)	289 (48)	60 (32)	178 (29)	7 (54)	20 (50)	2 (25)	4 (17)
Unknown^†^	**245 (61)**	**789 (62)**	104 (54)	308 (52)	129 (68)	441 (71)	6 (46)	20 (50)	6 (75)	20 (83)
**Total**	**402**	**1,280**	**192**	**597**	**189**	**619**	**13**	**40**	**8**	**24**

* The denominator for percentages of foods that reported recreational harvesting is the subtotal. The denominator for percentages of foods with unknown harvesting practice is the total.

† Unknown category does not include “not reported” because harvesting practice is not a variable for which data are systematically collected in the Foodborne Disease Outbreak Surveillance System.

**TABLE 6:** Number and percentage* of reported marine toxin outbreaks and outbreak-associated illnesses, by location of food preparation — Foodborne Disease Outbreak Surveillance System, United States, 2011–2023

Reported food preparation location	Total		Scombroid toxin		Ciguatoxin		Shellfish-associated toxins^†^		Other marine toxins^§^	
	No. of outbreaks (%)	No. of illnesses (%)	No. of outbreaks (%)	No. of illnesses (%)	No. of outbreaks (%)	No. of illnesses (%)	No. of outbreaks (%)	No. of illnesses (%)	No. of outbreaks (%)	No. of illnesses (%)
**Private home**	**193 (51)**	**614 (53)**	37 (20)	98 (19)	142 (80)	472 (81)	8 (73)	27 (79)	6 (86)	17 (85)
**Restaurant**	**147 (39)**	**411 (36)**	119 (66)	327 (64)	26 (15)	80 (14)	2 (18)	4 (12)	0 (—^¶^)	0 (—)
Sit-down dining restaurant	**130 (34)**	**360 (31)**	104 (57)	283 (55)	24 (13)	73 (13)	2 (18)	4 (12)	0 (—)	0 (—)
Fast-food restaurant	**12 (3)**	**34 (3)**	12 (7)	34 (7)	0 (—)	0 (—)	0 (—)	0 (—)	0 (—)	0 (—)
Other restaurant type	**5 (1)**	**17 (1)**	3 (2)	10 (2)	2 (1)	7 (1)	0 (—)	0 (—)	0 (—)	0 (—)
**Commercial location**	**18 (5)**	**47 (4)**	14 (8)	37 (7)	3 (2)	7 (1)	0 (—)	0 (—)	1 (14)	3 (15)
Grocery store	**10 (3)**	**27 (2)**	7 (4)	20 (4)	2 (1)	4 (1)	0 (—)	0 (—)	1 (14)	3 (15)
Fair, festival, or temporary mobile service	**5 (1)**	**12 (1)**	5 (3)	12 (2)	0 (—)	0 (—)	0 (—)	0 (—)	0 (—)	0 (—)
Seafood or farmer's market	**3 (1)**	**8 (1)**	2 (1)	5 (1)	1 (1)	3 (1)	0 (—)	0 (—)	0 (—)	0 (—)
**Institutional location**	**6 (2)**	**22 (2)**	4 (2)	14 (3)	2 (1)	8 (1)	0 (—)	0 (—)	0 (—)	0 (—)
Office or indoor workplace	**4 (1)**	**14 (1)**	2 (1)	6 (1)	2 (1)	8 (1)	0 (—)	0 (—)	0 (—)	0 (—)
School, college, or university	**2 (—)**	**8 (1)**	2 (1)	8 (2)	0 (—)	0 (—)	0 (—)	0 (—)	0 (—)	0 (—)
**Ship or boat**	**4 (1)**	**12 (1)**	1 (1)	4 (1)	3 (2)	8 (1)	0 (—)	0 (—)	0 (—)	0 (—)
**Hospital or other healthcare facility**	**3 (1)**	**9 (1)**	3 (2)	9 (2)	0 (—)	0 (—)	0 (—)	0 (—)	0 (—)	0 (—)
**Banquet or event facility**	**2 (—)**	**22 (2)**	2 (1)	22 (4)	0 (—)	0 (—)	0 (—)	0 (—)	0 (—)	0 (—)
**Other**	**4 (1)**	**12 (1)**	1 (1)	3 (1)	2 (1)	6 (1)	1 (9)	3 (9)	0 (—)	0 (—)
**Single location**	**377 (94)**	**1,149 (90)**	181 (94)	511 (86)	178 (94)	581 (94)	11 (85)	34 (85)	7 (88)	20 (83)
**Multiple locations**	**8 (2)**	**80 (6)**	3 (2)	59 (10)	5 (3)	21 (3)	0 (—)	0 (—)	0 (—)	0 (—)
**Location not reported or unknown**	**17 (4)**	**51 (4)**	8 (4)	24 (4)	6 (3)	17 (3)	2 (15)	6 (15)	1 (13)	4 (17)
**Total**	**402**	**1,280**	**192**	**597**	**189**	**619**	**13**	**40**	**8**	**24**

* The denominator for the location percentages is the total for single locations reported. The denominator for percentages for the single locations, multiple locations, and locations not reported or unknown is the total.

^†^ Of the six paralytic shellfish poisoning outbreaks, four resulted from food prepared in private homes and two from food prepared in unknown locations. Of the four neurotoxic shellfish poisoning outbreaks, all resulted from foods prepared in private homes. One amnesic shellfish poisoning outbreak resulted from food prepared in a sit-down dining restaurant. One unknown shellfish poisoning outbreak resulted from food prepared in a sit-down dining restaurant. One diarrhetic shellfish poisoning outbreak resulted from food prepared in another location.

^§^ Of the four outbreaks resulting in Haff disease, two resulted from food prepared in private homes, one from food prepared in a grocery store, and one from food prepared in an unknown location. Three puffer fish tetrodotoxin outbreaks resulted from food prepared in private homes. One unknown fish poisoning outbreak resulted from food prepared in a private home.

^¶^ Dashes indicate percentage values <1%.

### Scombroid Toxin

Scombroid toxin caused 192 outbreaks during 2011–2023 (182 confirmed and 10 suspected), resulting in 597 illnesses and six hospitalizations (Table 1). The median number of outbreaks per year was 13 (range = 8–25) (Supplementary Figure 2). The median number of illnesses per outbreak was two (range = 2–50). New York (43 outbreaks and 103 illnesses) and Florida (38 outbreaks and 93 illnesses) reported the most outbreaks and illnesses, followed by California (23 outbreaks and 64 illnesses) and Hawaii (18 outbreaks and 54 illnesses) (Table 2). One multistate outbreak caused by contaminated yellowfin tuna resulted in 50 (8%) illnesses across 11 states. Of the 192 outbreak reports, 189 (98%) included an implicated food, of which tuna was implicated in 144 outbreaks (76%) (Table 3). Of 131 outbreak reports with known food importation status, 70 (53%) implicated imported food (Table 4). None of the implicated foods were recreationally harvested (Table 5). Among the 181 (94%) outbreak reports that identified a single location of food preparation, sit-down dining restaurants were reported for 104 outbreaks (57%) (Table 6).

### Ciguatoxin

Ciguatoxin caused 189 outbreaks (174 confirmed and 15 suspected), resulting in 619 illnesses and 67 hospitalizations (Table 1). The median number of outbreaks per year was 15 (range = 3–31) (Supplementary Figure 3). The median number of illnesses per outbreak was three (range = 2–14). Most outbreaks and illnesses were reported by Florida (88 outbreaks and 274 illnesses), Puerto Rico (55 outbreaks and 185 illnesses), and Hawaii (18 outbreaks and 54 illnesses) (Table 2). Of the 189 outbreak reports, 187 (99%) identified an implicated food. Barracuda was implicated in 58 (31%) of these outbreaks, grouper in 25 (13%), and amberjack in 22 (12%) (Table 3). Of the 164 outbreak reports with an importation status, 142 (87%) implicated domestically caught fish (Table 4). Of the 60 outbreak reports with information about how implicated fish were sourced, 36 (60%) indicated that the implicated fish were recreationally harvested (Table 5). Of the 178 outbreak reports that identified a single location of preparation, private homes were reported for 142 outbreaks (80%) (Table 6).

### Shellfish-Associated Toxins

Shellfish-associated toxins caused six outbreaks of paralytic shellfish poisoning (23 illnesses and four hospitalizations), four outbreaks of neurotoxic shellfish poisoning (10 illnesses and three hospitalizations), one outbreak of amnesic shellfish poisoning (two illnesses and two hospitalizations), one outbreak of diarrhetic shellfish poisoning (three illnesses), and one outbreak of an unknown shellfish toxin (two illnesses) (Table 1). The median number of outbreaks per year was one, ranging from zero to three (Supplementary Figure 4). The median number of illnesses per outbreak was three, ranging from two to seven. Of the 13 outbreaks caused by shellfish-associated toxins, Florida reported five (38%) outbreaks and Alaska reported four (31%) outbreaks (Table 2). All outbreak reports implicated a known food source, with mussels in four (31%), sea snails in four (31%), and clams in three (23%) (Table 3). One outbreak investigation implicating paralytic shellfish poisoning identified puffer fish as the food source. Among the 11 outbreak reports that included importation status, all implicated domestically caught shellfish (Table 4). Of the seven outbreak reports with information about how implicated shellfish were sourced, six (86%) indicated that the implicated shellfish were recreationally harvested (Table 5). A single location of preparation was reported in 11 (85%) outbreaks, of which 73% were associated with shellfish prepared in a private home (Table 6).

### Other Marine Toxins

Eight outbreaks caused by three other marine toxins were reported during 2011–2023: four by an unidentified toxin associated with Haff disease (11 illnesses, 10 hospitalizations, and one death), three by puffer fish tetrodotoxin (nine illnesses and four hospitalizations), and one by an unspecified fish toxin (four illnesses) (Table 1). The number of outbreaks per year ranged from zero to two (Supplementary Figure 5). Implicated foods were reported for seven outbreaks and included puffer fish in three outbreaks (43%), buffalo fish in two outbreaks (29%), roe in one outbreak (14%), and carp in one outbreak (14%) (Table 3). Six outbreak investigations (86%) implicated fish that were prepared in a private home (Table 6). Domestically caught fish were implicated in five outbreaks (71%) (Table 4).

## Discussion

Scombroid toxin, ciguatoxin, and shellfish-associated toxins were responsible for nearly all outbreaks and associated illnesses and hospitalizations from marine toxins in the United States during 2011–2023. The high levels of histamine that cause scombroid toxin poisoning accumulate as a result of improper temperature control of seafood, which can occur at any point from harvest to consumption, including during transport. A better understanding of the practices throughout the supply chain might provide an opportunity to identify food safety risks and provide recommendations to help prevent scombroid toxin outbreaks. More than one half of scombroid toxin outbreak reports implicated fish imported from outside the United States, highlighting the need for continued efforts to ensure safe handling practices for imported fish. Although food safety and temperature control standards for restaurants exist to prevent scombroid toxin poisoning, >60% of outbreak reports identified restaurants as the location where the implicated food was prepared. These findings suggest that more attention to food safety practices before and at the point-of-sale in restaurants that serve seafood commonly associated with scombroid toxin poisoning might be needed.

In contrast with scombroid toxin poisoning, foodborne illnesses from ciguatoxin and shellfish-associated toxins occur by eating fish and shellfish contaminated with algal toxins in areas where toxin-producing algae grow (*5*,*6*). Standard food safety practices that limit microbial contamination, survival, and proliferation in the food product do not reduce the risk for illness associated with foodborne algal toxins (*21*). Thus, interventions should incorporate a collaborative, multisectoral, and transdisciplinary One Health approach. Intervention activities could include environmental monitoring (e.g., water sampling and satellite data) to detect algal blooms or marine toxins in water or seafood before harvest. Reducing harvesting of reef fish and shellfish from high-risk areas, especially during and immediately after harmful algal blooms, might prevent illnesses from these toxins (*22*).

Nearly all outbreaks caused by ciguatoxin were reported by Florida, Puerto Rico, and Hawaii, where algal species that produce ciguatoxin are endemic. Most of the outbreak investigations in these states implicated reef fish that were not imported and that were prepared in a private home. Although information about how fish were caught is not systematically collected in FDOSS, many outbreak reports from these states indicated that the implicated fish were recreationally caught. This finding suggests that recreational fishing might be a driver of ciguatoxin outbreaks in endemic states. Better understanding of the practices of recreational fishers might provide opportunities for education about ciguatoxin poisoning prevention. Toxins produced by certain *Gambierdiscus* species are the primary cause of ciguatoxin poisonings in the United States and the geographic range of these algae has expanded (*7*). More comprehensive data about recreational harvesting locations, algal species’ geographic range, and frequency of algal bloom occurrence could guide longer-term understanding of risks associated with eating fish species known to bioaccumulate algal toxins.

Outbreaks caused by ciguatoxin were also reported by several states where ciguatoxin-producing algal species are not endemic, and a large majority of these outbreaks involved domestically caught reef fish not typically found in the wild in those states. Educating both fish processors and consumers in these states about ciguatoxin, the affected fish species, and endemic areas might reduce exposure. Outreach to processors and consumers that accurately reflects knowledge about potential risks might be needed in both endemic and nonendemic areas.

Although reported less frequently, outbreaks of shellfish-associated toxins were more severe than outbreaks of other toxins, with 25% of illnesses resulting in hospitalizations. Paralytic shellfish poisoning and neurotoxic shellfish poisoning caused >75% of outbreaks involving shellfish-associated toxins. Commercially harvested shellfish routinely undergo laboratory testing for toxins that cause paralytic and neurotoxic shellfish poisoning (*23*,*24*); paired with other prevention measures, testing has reduced poisonings from commercial sources (*6*). Non–laboratory-based tests for detecting shellfish toxins do not exist, so recreationally harvested shellfish do not undergo such testing. However, many state health departments have state-managed programs that regularly monitor and test shellfish from public shorelines and can provide updated shellfish safety maps. All outbreaks associated with paralytic and neurotoxic shellfish poisoning were reported by Alaska, Florida, and Washington, where recreational shellfish harvesting is common, and nearly all outbreaks involved domestically harvested clams, mussels, and sea snails that were prepared in a private home. Furthermore, many outbreak reports indicated that the implicated shellfish were recreationally harvested. This observation suggests that recreational harvesters are an important target population for prevention, for whom tailored communications and education on state-managed monitoring programs for marine toxins could help reduce the occurrence of outbreaks. In addition, multidisciplinary collaboration can strengthen responses to lesser-known food sources of shellfish toxins, such as marine snails in Florida (*25*).

## Limitations

The findings in this report are subject to at least three limitations. First, the number of marine toxin outbreaks reported to CDC via FDOSS is likely an underestimate because of underascertainment of cases. No readily available diagnostic tests for marine toxins in humans exist, and clinical diagnosis typically relies on food history and a combination of complex symptoms which could lead to misdiagnosis. In addition, many health care professionals are unaware that foodborne outbreaks from marine toxins are reportable and therefore might not notify public health departments when they occur (*26*). In places where poisonings from marine toxins are common, ill persons might not seek medical care because they are familiar with the poisonings (*27*). Furthermore, the response to COVID-19 had a substantial effect on the time and resources of state and local health departments, which likely diminished the ability to investigate and report outbreaks to FDOSS during 2020–2022 (*28*). Second, assessing trends over time is difficult because they might represent changes in outbreak reporting rather than a true increase or decrease in the number of outbreaks in a geographic location. Finally, FDOSS collects data on outbreaks, limiting conclusions that can be drawn about case-level demographic characteristics and sporadic illnesses caused by marine toxins.

## Future Directions

Prevention of scombroid toxin outbreaks could be enhanced by ensuring adequate temperature control of seafood from catch to consumption. Traceback investigations of outbreaks implicating imported fish could provide insight into opportunities for public health guidance (*29*). Improved data collection of food safety practices in restaurants linked to outbreaks, such as adherence to temperature control practices from receipt of food to preparation or employee training on proper fish handling procedures, could help identify lapses in restaurant food safety. Prevention of foodborne disease outbreaks associated with algal toxins requires continued measures such as commercial harvest monitoring and targeted communications about safer harvesting practices to affected populations, especially in endemic areas. Public health messaging can be tailored to the needs of recreational fishers in areas where toxin-producing algal species are endemic. In addition, CDC could consider collecting data in FDOSS about how (recreationally or commercially) and where implicated fish and shellfish are harvested to better guide public health messaging.

## Conclusion

Surveillance of foodborne disease outbreaks associated with marine toxins can guide prevention efforts. Scombroid toxin poisoning prevention relies on ensuring adequate temperature control of seafood; a better understanding of food safety practices for imported fish and food handlers in restaurants is critical for prevention efforts. Most outbreaks caused by ciguatoxin and shellfish-associated toxins implicated recreationally harvested seafood. Targeted messaging about affected areas and species is essential for prevention of outbreaks from algal toxins linked to recreational fishing.

## Footnotes

* These authors contributed equally to this work.

† These senior authors contributed equally to this work.

§ 45 C.F.R. part 46.102(l)(2), 21 C.F.R. part 56; 42 U.S.C. Sect. 241(d); 5 U.S.C. Sect. 552a; 44 U.S.C. Sect. 3501 et seq.
